# Mastication

**Published:** 1916-11

**Authors:** C. M. M’Cauley

**Affiliations:** Dallas, Texas


### Mastication.

BY C. M. M'CAULEY, B. S., D. D. S., DALLAS, TEXAS.

The principal function of the dental apparatus is mastication of food. Animals that masticate food are divided into three distinct classes, namely, carnivorous, herbivorous, and a combination of these two. Observation teaches us that there are marked differences in the masticatory organs of these different classes. For example, the cow, belonging to the herbivorous class, has molars only. The dog, belonging to the carnivorous class, has no molars; and man, the combination of the two classes, has a combination dental apparatus which is similar to a combination denture of the dog and the cow.

In order that assimilation may be perfect, vegetable foods or starches must be thoroughly masticated. The cell walls must be broken down. This requires molars, or grinding teeth. The cow masticates well because it is necessary for the digestion of the food she eats. The dog swallows his food whole and the stomach bears the burden of breaking up the food as well as digesting it.

All this simply means that the molars in a man's mouth prepare him for proper mastication and assimilation of starches, and when his molars are lost his ability to digest starches is greatly impaired. The person who has lost his back teeth should lose no time in having them replaced by artificial means. In cases where there are no teeth for grinding and breaking up the starch cells the meat diet will be found more easily digested.

Thorough mastication, especially of starchy foods, cannot be too strongly urged. A good dental apparatus is essential to good mastication. Loss of a tooth or teeth, a cavity in a tooth producing a lameness, or sensitive tooth surfaces or inflamed gums conduce to imperfect mastication. Imperfect mastication means impaired digestion and impaired digestion is followed by a reduced vitality and a lowered opsonic index. With these conditions to deal with, saying nothing of the various kinds of microorganisms present in the mouth when any or all of these conditions are present, the physician is greatly handicapped in his efforts to restore health. In considering the requirements for health or the causes of disease; the source of strength or the origin of weakness; the preservation of life and the restoration of health, when we look intelligently for the basis of it all we shall find the dental apparatus playing a greater or lesser part, more often the greater part. Because through the mouth and adjacent organs pass the elements which provide all life giving properties.

The mouth conditions mentioned above which impair mastication and digestion are produced by neglect and ignorance more than anything else. Neglect on the part of the physician to discover and remove adenoids in the early years of the child's life, as mentioned in the preceding paper, causes the raising of the vault, narrowing of the upper arch and destroys the occlusion of the upper and lower arches of teeth. The failure of the physician to look into the child's mouth and discover decayed teeth and have them repaired will result in a similar tragedy in the life of the child. Failure of the physician to insist upon a clean mouth may result in decay of the teeth, producing a lameness if not loss of the teeth. Either of these impair mastication. Failure of the physician to advise patient to seek dental advice may result in pyorrhea with all its deadly consequences. These things and many others too numerous to mention here seem to make it evident that the physician should make the mouth condition not only an index to be used in every diagnosis of diseased conditions, but it should never be overlooked in his efforts to guard the health of his patients. When I say that physicians everywhere should regard mouth conditions as taking a primary place in the causation of disease you will all agree with me. Then when I ask why this has not and is not being done, the only answer can be *negligence.*

The tendency of the dental profession today is toward prevention of conditions which produce faulty mastication. Caries of teeth is preventable; pyorrhea is preventable; irregularities of the teeth are preventable. Intelligent and strict observation on the part of the physician coupled with efficient and prompt action on the part of the dentist will accomplish the result.

---

The cause, ills, and prevention of dental caries and pyorrhea will be considered in future papers.

